# Dasatinib Treatment Increases Sensitivity to c-Met Inhibition in Triple-Negative Breast Cancer Cells

**DOI:** 10.3390/cancers11040548

**Published:** 2019-04-17

**Authors:** Patricia Gaule, Nupur Mukherjee, Brendan Corkery, Alex J. Eustace, Kathy Gately, Sandra Roche, Robert O’Connor, Kenneth J. O’Byrne, Naomi Walsh, Michael J. Duffy, John Crown, Norma O’Donovan

**Affiliations:** 1 Molecular Therapeutics for Cancer Ireland, National Institute for Cellular Biotechnology, Dublin City University, Dublin D09 NR58, Ireland; Patricia.gaule@yale.edu (P.G.); mukherjeen@nirrh.res.in (N.M.); bcorkery@svhg.ie (B.C.); sandra.roche@dcu.ie (S.R.); roconnor@irishcancer.ie (R.O.); Naomi.walsh@dcu.ie (N.W.); john.crown@ccrt.ie (J.C.); normaodonov@gmail.com (N.O.); 2 Trinity Translational Medicine Institute, St. James’s Hospital Dublin, Dublin 8, Ireland; gatelyk@tcd.ie; 3 Institute of Health and Biomedical Innovation, Queensland University of Technology, Translational Research Institute, Woolloongabba QLD 4059, Australia; k.obyrne@qut.edu.au; 4 UCD School of Medicine, UCD Conway Institute of Biomolecular and Biomedical Research, University College Dublin, Dublin 4, Ireland; michael.J.Duffy@ucd.ie; 5 UCD Clinical Research Centre, St. Vincent’s University Hospital, Dublin 4, Ireland; 6 Department of Medical Oncology, St Vincent’s University Hospital, Dublin 4, Ireland

**Keywords:** Src kinase, basal-like breast cancer, cMet

## Abstract

In pre-clinical studies, triple-negative breast cancer (TNBC) cells have demonstrated sensitivity to the multi-targeted kinase inhibitor dasatinib; however, clinical trials with single-agent dasatinib showed limited efficacy in unselected populations of breast cancer, including TNBC. To study potential mechanisms of resistance to dasatinib in TNBC, we established a cell line model of acquired dasatinib resistance (231-DasB). Following an approximately three-month exposure to incrementally increasing concentrations of dasatinib (200 nM to 500 nM) dasatinib, 231-DasB cells were resistant to the agent with a dasatinib IC_50_ value greater than 5 μM compared to 0.04 ± 0.001 µM in the parental MDA-MB-231 cells. 231-DasB cells also showed resistance (2.2-fold) to the Src kinase inhibitor PD180970. Treatment of 231-DasB cells with dasatinib did not inhibit phosphorylation of Src kinase. The 231-DasB cells also had significantly increased levels of p-Met compared to the parental MDA-MB-231 cells, as measured by luminex, and resistant cells demonstrated a significant increase in sensitivity to the c-Met inhibitor, CpdA, with an IC_50_ value of 1.4 ± 0.5 µM compared to an IC_50_ of 6.8 ± 0.2 µM in the parental MDA-MB-231 cells. Treatment with CpdA decreased p-Met and p-Src in both 231-DasB and MDA-MB-231 cells. Combined treatment with dasatinib and CpdA significantly inhibited the growth of MDA-MB-231 parental cells and prevented the emergence of dasatinib resistance. If these in vitro findings can be extrapolated to human cancer treatment, combined treatment with dasatinib and a c-Met inhibitor may block the development of acquired resistance and improve response rates to dasatinib treatment in TNBC.

## 1. Introduction

Triple-negative breast cancer (TNBC) lacks expression of estrogen receptor (ER) and progesterone receptor (PR) and exhibits overexpression of human epidermal growth factor receptor 2 (HER2). It accounts for approximately 15% of all breast cancer cases and patients with this form demonstrate higher rates of recurrence and shorter disease progression than those with luminal or HER2-positive tumours [1]. Treatment of TNBC predominantly relies on the use of cytotoxic chemotherapies due to a lack of proven molecular targets [2].

We have previously shown that Proto-oncogene tyrosine-protein kinase Src (Src kinase) is frequently expressed in TNBC and may be a rational therapeutic target for TNBC [3]. Src is a proto-oncogene and a member of the Src family kinases (SFKs). SFKs are non-membrane-bound tyrosine kinases consisting of nine members. Src, Yes, Fyn and Fgr belong to the Src A family subtype. Lck, Hck, Blk and Lyn belong to Src B family subtype, with Frk in its own subfamily [4]. The SFKs display high levels of homology to one another and are involved in propagating downstream cell signalling to effect cellular biological functions including cell proliferation, cell migration, angiogenesis and cell survival [5,6,7].

Dasatinib is a multi-targeted tyrosine kinase inhibitor whose targets include BCR/Abl and Src family kinases. Pre-clinical studies suggested that basal-like TNBC cell lines show higher sensitivity to dasatinib than luminal cell lines, supporting dasatinib as a potential targeted treatment for TNBC [3,8,9]. Additionally, when TNBC was further sub-classified into six molecular types, cell line models of the mesenchymal and mesenchymal stem-like group showed greater sensitivity to dasatinib than other cell line models [3,9,10]. However, in a phase II clinical trial, dasatinib showed limited single-agent activity in patients with metastatic breast cancer [11] or in patients with locally advanced or metastatic TNBC [12].

Several potential predictive biomarkers of response to dasatinib treatment have been identified [13], and a prospective phase II trial was designed to test three predictive gene signatures, whereby patients with metastatic breast cancer whose metastatic tumour biopsies were positive for one of the gene signatures were treated with dasatinib [13]. However, no significant clinical benefit was observed despite selecting patients having the highest scores for the three predictive signatures.

Thus, despite promising activity in preclinical studies, dasatinib has so far failed to produce clinical benefits in TNBC patients. One of the limitations of the preclinical studies that showed activity in TNBC cells, including our own [3], is that they were limited to short-term proliferation assays. Therefore, we examined the effects of longer exposure to dasatinib on TNBC cells that are highly sensitive to dasatinib in short-term proliferation assays. The overall aim was to identify novel combinatorial approaches that may block the development of acquired resistance to dasatinib and improve response rates to dasatinib treatment in TNBC.

## 2. Materials and Methods

### 2.1. Cell Lines and Reagents

MDA-MB-231 cells were obtained from the American Type Culture Collection (ATCC). 231-DasB was developed by continuous exposure to dasatinib (Sequoia Research Products, Berkshire, UK) with a starting concentration of 200 nM, increasing incrementally to a maximum of 500 nM dasatinib over a period of 13 weeks. Untreated (parental) MDA-MB-231 cells were cultured in a similar manner in the absence of drug to create an aged-matched control. Both cell lines were cultured in RPMI 1640 with 10% Foetal Bovine Serum, plus 10 nM dasatinib for 231DasB. Stock solutions (10 mM) of PD180970 (Merck, Dublin, Ireland), elacridar (Sequoia Research Products, Berkshire, UK) and Compound A (CpdA, Amgen, Thousand Oaks, CA, USA) were prepared in DMSO.

### 2.2. Proliferation Assays

Proliferation was measured in triplicate biological assays using an acid phosphatase assay or viable cell count. For acid phosphatase assays, 1 × 10^3^ cells/well were seeded in 96-well plates. Plates were incubated overnight at 37 °C followed by the addition of the drug at the appropriate concentrations and incubated for a further five days until wells were 80–90% confluent. All media were removed and the wells were washed once with phosphate buffered solution (PBS). Paranitrophenol phosphate (PNP) substrate (Sigma-Aldrich, Dublin, Ireland) (10 mM of PNP in sodium acetate buffer, pH 5.5) was added to each well and incubated at 37 °C for 1 h. Fifty microliters of 1 M NaOH were added and the absorbance was read at 405 nM (reference: 620 nM). For viable cell counts, cells were trypsined and combined in a 1:1 ratio with Viacount Flex reagent and counted using a Guava Easycyte (Merck, Dublin, Ireland).

### 2.3. Doubling Time Assays

Next 2 × 10^3^ cells were seeded in 24-well plates in 1 mL of serum-containing medium. Fresh medium (control) or drug-containing medium was added to the cells. Assays were conducted in triplicate. Duplicate wells per cell line for each condition (control and drug treatments) were seeded for each time point: day 0, day 4, and day 7. Cell counts were measured using the Guava Viacount method. Doubling times were calculated between days 4 and 7 using the formula

$$\mathrm{Doubling}\;\mathrm{time}\;\left(\mathrm{hours}\right)=24\times \frac{\log2x\left(Tt-T0\right)}{\left(\mathrm{logNt}-\mathrm{logN}0\right)},$$

where Tt is the end time point and T0 is the beginning time point (days), which in this case were 7 and 4, respectively, and N is the average cell count on each day.

### 2.4. Short-Term Resistance Assay

For the short-term resistance assays, 1.5 × 10^4^ MDA-MB-231 cells per well were seeded in two identical 12-well plates (Plate-1 and Plate-2) in 1 mL of medium with 10% foetal bovine serum (day 1) and allowed to adhere overnight. The cells were treated with 2.5 µM compound A and/or 50 nM dasatinib. In addition, in plate 1, three wells were untreated as a control. In plate 2, the cells were similarly treated with 2.5 µM compound A and/or 50 nM dasatinib. Treatment was repeated twice weekly. After seven days, when the untreated control cells achieved confluency, the medium was removed from the cells in plate 1 and the cells were fixed with 3:1, v:v, methanol:acetic acid, (1 min), then washed with PBS and stained with 0.05%, w:v, crystal violet (5 min). The cells were allowed to air dry overnight, the crystal violet was eluted in 10% acetic acid and absorbance was measured at 590 nM. After 21 days, when the dasatinib-treated cells achieved confluency, cells in plate 2 were fixed and stained as for plate 1.

### 2.5. Invasion/Migration Assays

Invasion and migration assays were carried out using 5 × 10^4^ cells in Matrigel (Corning)-coated 24-well invasion inserts (BD Biosciences) for invasion assays and uncoated inserts for migration assays. Cells were seeded in reduced serum medium (5% foetal calf serum in RPMI-1640) and incubated for 6 h to allow cell attachment, and then treated with 100 nM dasatinib for 18 h. Cells were stained with crystal violet and the number of invading/migrating cells was estimated by counting 10 fields of view at 200× magnification. The average count was multiplied by the conversion factor 140 (growth area of membrane divided by field of view area, viewed at 200× magnification) to determine the total number of invading/migrating cells. Invasion and migration assays were carried out in triplicate.

### 2.6. Dasatinib Accumulation Assays

Cells were seeded at the specified numbers in T25-cm^2^ cell culture flasks (Thermo Fisher, Dublin, Ireland). After 24 h, the cell culture medium was removed and cells were treated with medium (control) or medium containing 2 µM dasatinib for 2 h. Non-adherent cells were collected and combined with adherent cells after trypsinisation, then centrifuged at 300 *g* for 3 min. The supernatant was removed and the cells were resuspended in 1 mL of ice-cold PBS. Fifty microliters of the cell suspension were removed for cell counting using the Guava Viacount method. The cell suspension was centrifuged as before, and the supernatant was removed. The cell pellet was stored at −20 °C.

Quantification of the mass of dasatinib present in the cells collected was performed using liquid chromatography tandem mass spectrometry (LC-MS/MS), as previously described [14]. Briefly, the drug was extracted using a liquid-liquid extraction procedure. One hundred microliters of 500 ng/mL lapatinib were added to an extraction tube (internal standard), along with 200 µL of 1 M Ammonium Formate pH 3.5 buffer and 1.6 mL of extraction solvent tert-Butyl Methyl Ether (tBME)/ acetonitrile (ACN) (3:1 v:v). The extraction tubes were vortexed and mixed on a blood tube mixer for 15 min. The samples were centrifuged at 6500 *g* for 5 min; the organic layer was removed and 1.1 mL of solvent was transferred to conical bottomed glass LC autosampler vials (Sigma-Aldrich, Dublin, Ireland). The vials were evaporated to dryness using a Genevac EZ-2 (Ipswich, UK) evaporator at ambient temperature, without light. The samples were reconstituted in 40 µL of acetonitrile with 20 µL injected automatically by the autosampler. The LC-MS was run in isocratic mobile phase (54% ACN:10 mM ammonium formate, pH 4) on a Hyperclone BDS C18 column, in multi-reaction monitoring (MRM) positive ion mode. Analysis was performed using MRM mode with the following transitions: m/z 581→m/z 365 for lapatinib, and m/z 488→m/z (231 and 401) for dasatinib, with a dwell time of 200 ms. Quantification was based on the integrated peak area determined by the Masshunter Quantification Analysis software, which quantitates the peak areas of the MRM transitions of each analyte. Results are reported as mean and standard deviation (SD) of the mass per million cells in triplicate flasks.

### 2.7. Protein Extraction and Western Blotting

RIPA buffer with 1× protease inhibitors, 2 mM PMSF and 1 mM sodium orthovanadate (Sigma-Aldrich) was added to cells and incubated on ice for 20 min. Following centrifugation at 10,000 rpm for 10 min at 4 °C, the resulting lysate was stored at −80 °C. Protein quantification was performed using the bicinchoninic acid (BCA) assay (Pierce). Thirty micrograms of protein in sample buffer were heated to 95 °C for 5 min and proteins were separated on 7.5% gels (Lonza) or 4–12% gels (Thermo-Fischer, Dublin, Ireland). The protein was transferred to a nitrocellulose membrane. The membrane was blocked with a blocking solution (PBS + 0.1% Tween + 5% skimmed milk powder (BioRad, Dublin, Ireland) or a 1× NET solution buffer (0.5 M NaCl, 0.05 M EDTA, 0.1 M Tris pH 7.8) at room temperature for 1 h, then incubated overnight at 4 °C in a blocking solution with a 1:1000 antibody dilution of total Src, c-Met, p-Met Y1234/1235 (Cell Signalling Technology, Leiden, Netherlands) or 1:500 p-Src Y419 (Merck-Millipore). The membrane was washed three times with PBS-Tween or 1× NET, then incubated at room temperature with anti-mouse secondary antibody (Sigma-Aldrich) at 1:1000 dilution or anti-rabbit secondary antibody (Sigma-Aldrich) at a 1:1000 dilution in blocking solution for 1 h. The membrane was washed three times with PBS-Tween/1× NET followed by one PBS wash. Detection was performed using Luminol (Santa Cruz Biotechnology, Heidelberg, Germany).

### 2.8. Luminex Magnetic Bead Assays

Magnetic bead assays were performed on the Luminex^®^ MagPix^®^ System (Merck Millipore (80-073), Dublin, Ireland) using Milliplex Map Phospho Mitogenesis RTK Magnetic Bead 7-Plex Kit (Merck Millipore 48-672 Mag) and Milliplex Map phospho Human Src Family Kinase Magnetic Bead 8-Plex kit (Merck Millipore 48-650 Mag). Protein extractions were prepared as described above. Protein (1–10 µg) was diluted in appropriate volume of assay buffer (final volume: 25 µL/well) and the assay was performed as per the manufacturer’s instructions.

### 2.9. DNA Extraction and Nested PCR Amplification of Src Exons 9–12

DNA was extracted from the parent MDA-MB-231 and the resistant 231DasB cell lines using the QIAamp^®^ DNA mini kit (Qiagen, Hilden, Germany). Exons 9–12 of the *Src* gene were amplified using the primer sets in Table S1. The forward outer primer and the reverse primer were used in the first PCR reaction and the forward inner primer and the reverse primer were used in the second PCR reaction. The following PCR conditions were used for the first reaction: 5 µL 10× Amplitaq Gold Buffer (Thermo-Fischer, Dublin, Ireland), 3 µL 25 mM MgCl_2_, 1 µL 10 mM dNTPs, 5 µM each of forward and reverse primer, 0.25 µL Amplitaq Gold DNA Polymerase (Applied Biosystems, Foster City, CA, USA) and 50 ng of DNA made up to a volume of 50 µL with dH_2_O. A pre-PCR heat step of 95 °C for 5 min was carried out to activate the enzyme and the DNA was amplified for 35 cycles at 95 °C (1 min), 56 °C (1 min) and 72 °C (1 min) and at 72 °C (10 min) after the last cycle. The second PCR was carried out as above with 1 µL of the first PCR reaction replacing the DNA. Ten microliters of the PCR product were electrophoresed on 1% agarose gel to verify product integrity. PCR products were purified using a QIAquick PCR purification kit (Qiagen). The DNA concentration was measured using the Nanodrop 1000 spectrophotometer (Thermo Fischer, Dublin, Ireland).

### 2.10. Cycle Sequencing of PCR Products

Cycle sequencing reactions were set up as follows: 2 µL of BigDye^®^ Terminator Mix v3.1, 20 ng amplicon DNA, 3.2 pmol of forward or reverse primer, 2 µL sequencing buffer and diluted to 20 µL with water. A positive control was also set up to ensure the efficiency of the sequencing reaction (1 µL pGem, 2 µL M13 primer, 2 µL of BigDye^®^ Terminator Mix v3.1 and 2 µL sequencing buffer). The pGem and BigDye^®^ Terminator v3.1 mix were both sourced from Applied Biosystems (Warrington, UK). Initial denaturation was carried out by a rapid thermal ramp to 96 °C (1 min), followed by 25 cycles of: rapid thermal ramp to 96 °C (10 s), rapid thermal ramp to 50 °C (5 s), rapid thermal ramp to 60 °C (4 min). Unincorporated dye terminators were removed before performing capillary electrophoresis using the DyeEx 2.0 Spinkit (Qiagen). Sequencing was performed on a 3130xl genetic analyser (Thermo-Ficsher, Ireland, and sequencing files were analysed using the BioEdit v 7.0.8 (Tom Hall, Ibis Biosciences, Carlsbad, CA, USA).

### 2.11. Statistical Analysis

Alterations in doubling times of cell lines, changes in the invasive and migratory potential of cell lines, and alterations in proteomic signalling were measured using the Student’s *t*-test. Error bars represent the standard deviation of triplicate experiments, where ‘*’/’**’indicates a *p*-value of ≤ 0.05/0.01, respectively, where a *p*-value < 0.05 was considered statistically significant.

## 3. Results

### 3.1. Dasatinib Exposure Induces a Resistant Phenotype

**Figure 1:**
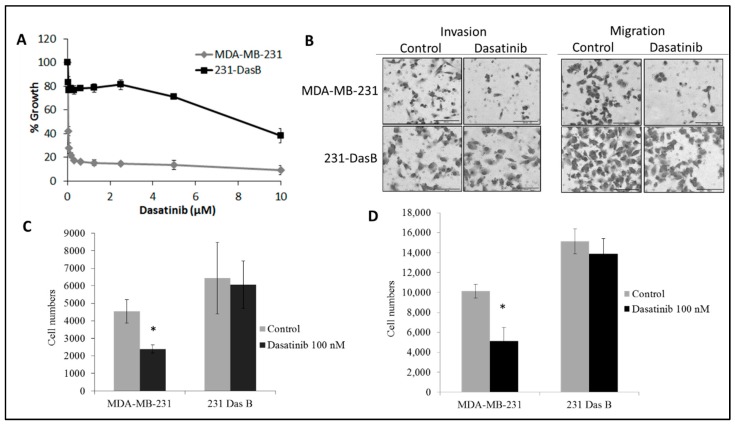
Characterisation of the 231DasB cell line: (**A**) Proliferation assays of MDA-MB-231 and 231-DasB with serially decreasing concentrations of dasatinib from 10 µM; (**B**) representative images of MDA-MB-231 and 231-DasB post-18 h 100 nM dasatinib treatment in invasion and migration assays; (**C**) invasion assays and (**D**) migration assays of MDA-MB-231 and 231-DasB post-18 h 100 nM dasatinib treatment. Error bars represent the standard deviation of triplicate experiments. * *p* ≤ 0.05. *p* values were calculated using the Student’s *t*-test.

MDA-MB-231 cells are very sensitive to growth inhibition by dasatinib (IC_50_ = 40 ± 1 nM) [3]. A cell line model of acquired resistance to dasatinib, MDA-MB-231 cells, was developed by continuous exposure to dasatinib for approximately three months. Following this treatment the IC_50_ value for dasatinib increased to greater than 5 µM, confirming acquired resistance to dasatinib (IC_50_ > 1 µM) (8) compared to MDA-MB-231 cells (Figure 1A). The MDA-MB-231 cells that developed acquired resistance to dasatinib were referred to as 231 DasB.

No significant difference in growth rate was observed between the MDA-MB-231 parental cells and the 231-DasB cells. MDA-MB-231 cells show a significant dose dependent increase in doubling time in the presence of dasatinib, whereas the 231-DasB cells show no significant change in doubling time in response to dasatinib (Table 1).

We have previously shown that dasatinib treatment significantly decreases migration and invasion of MDA-MB-231 cells [3]. Migration, but not invasion, was significantly increased in the 231-DasB variant compared to the parental cell line (*p* = 0.009) (Figure 1B). In the 231-DasB-resistant variant, dasatinib did not inhibit invasion (*p* = 0.772) (Figure 1C) or migration (*p* = 0.340) (Figure 1D).

Dasatinib is a substrate for the drug efflux pumps BCRP and MDR-1 [15]. Therefore, in order to determine if drug efflux pumps were involved in the resistance to dasatinib in 231-DasB cells, we assessed the uptake of dasatinib in the parental MDA-MB-231 cells and the resistant variant, 231-DasB. Initially, dasatinib accumulation was measured using 2 µM dasatinib across a range of cell seeding numbers. While the relative mass of drug measured increased with decreasing cell density, no significant difference in accumulation between parental and variant cells was observed, suggesting drug pumps do not influence resistance to dasatinib in the 231-DasB cell line (Figure S1). To confirm this, we tested growth inhibition with dasatinib combined with the potent BCRP and MDR-1 inhibitor, elacridar [16]. Proliferation was measured in the parental and variant cell lines treated with dasatinib alone or in combination with a concentration of elacridar sufficient to inhibit BCRP [17] and MDR-1 [18]. The addition of elacridar did not cause a significant increase in dasatinib-induced growth inhibition in either the parental or the variant cell line (Figure S2).

### 3.2. Phosphorylation of Src Is Altered in Dasatinib-Resistant Cells

**Figure 2:**
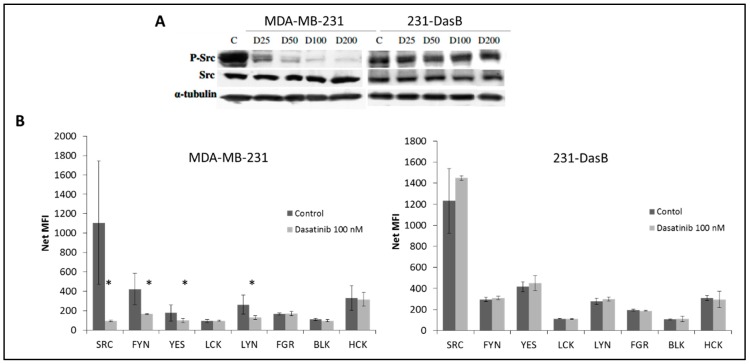
(**A**) Levels of total Src and phosphorylated Src [pY419] in MDA-MB-231 (parental and 231-DasB cells treated with dasatinib for 6 h. (P-: phospho-; C: control (untreated); D: dasatinib (nM)). (**B**) Phosphorylation of Src family kinases as determined by multiplex bead assay in MDA-MB-231 and 231-DasB cell lines with and without 6-h dasatinib treatment (100 nM). NET MFI is net median fluorescence intensity. Error bars represent the standard deviations of triplicate independent experiments. * indicates *p* < 0.05 calculated using the Student’s *t*-test.

The 231-DasB cells were tested for cross-resistance to the Src kinase inhibitor PD180970. The 231-DasB cells showed a 2.2-fold increase in the PD180970 IC_50_ compared to the MDA-MB-231 cells (876.1 ± 74.4 versus 400.2 ± 35.6 nM, *p* = 0.003), implicating Src in the resistant phenotype. Therefore, we examined phosphorylation of Src in the resistant cells. As expected, dasatinib treatment reduced the levels of p-Src (Y419) in MDA-MB-231 cells but no reduction in p-Src (Y419) was observed in the 231-DasB cells following dasatinib treatment (25–200 nM) (Figure 2A). The Src family kinases (SFKs) consist of nine family members with high conservation between family members at tyrosine 419 (Y419). We examined the phosphorylation of seven of these proteins using magnetic multiplex assays. Consistent with the Western blot results, MDA-MB-231 showed a significant reduction in p-Src in response to dasatinib treatment (Figure 2B). p-FYN, p-YES and p-LYN were also significantly reduced after treatment with dasatinib. In the 231-DasB cells, treatment with dasatinib did not decrease the level of any of the phospho-SFK proteins examined (Figure 2B).

To determine if a mutation in the active site of Src kinase may be causing constitutive activation of p-Src, in the presence of dasatinib, we sequenced exons 9–12 of the *Src* gene, which encompass tyrosine 419 and the regulatory site at tyrosine 530 [19,20], in both the MDA-MB-231 parental and 231DasB cells. No alteration in the Src sequence was detected in the 231DasB cells (Figure S3).

### 3.3. cMet Signalling Is Increased in Dasatinib-Resistant Cells

**Figure 3:**
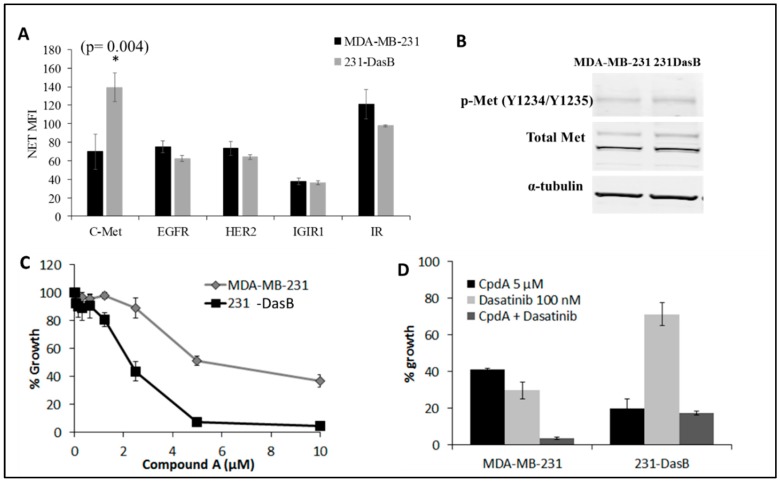
(**A**) Phosphorylation of five RTKs in the MDA-MB-231 and 231-DasB cell lines, where NET MFI is net median fluorescence. Error bars represent the standard deviations of triplicate independent experiments. * indicates *p* < 0.05. *p* values were calculated using the Student’s *t*-test. (**B**) Immunoblots for p-Met (Y1234/Y1235) and total Met in MDA-MB-231 and 231-DasB cells. α-tubulin was used a loading control. (**C**) MDA-MB-231 and 231-DasB dose-response curves with serially decreasing concentrations of CpdA from 10 µM; (**D**) Fixed concentration proliferation assays with 100 nM dasatinib and 5 µM CpdA in MDA-MB-231 and 231-DasB cells.

To investigate the possible role of upstream receptor tyrosine kinases (RTKs) in activation of Src kinase in the 231DasB cells, we examined the phosphorylation of a panel of RTKs using a multiplex assay. c-Met (panTYR) was the only RTK of the five tested that was altered in the 231-DasB cells. 231-DasB cells show significantly higher levels of p-Met (*p* = 0.004) as determined by ELISA, compared to the MDA-MB-231 cells (Figure 3A). We then demonstrated a non-significant 1.4-fold increase (*p* = 0.08) in phosphorylation of c-Met at its activation site (Y1234/Y1235) (Figure 3B). The difference in results between ELISA and Western blotting is likely due to the ELISA assay detecting all changes in p-Met activation, whilst we only analysed changes in p-Met (Y1234/1235) using Western blotting.

Based on the increased levels of p-Met in the 231-DasB cells, we examined sensitivity to a c-Met inhibitor, compound A (CpdA). The 231-DasB cells are more sensitive to CpdA, with an IC_50_ of 1.4 ± 0.5 µM, compared to the MDA-MB-231 cells where the CpdA IC_50_ is 6.8 ± 0.2 µM (Figure 3C).

Combined treatment with CpdA and dasatinib resulted in significantly decreased growth in MDA-MB-231 (*p* = 0.02) (Figure 3D). In the 231-DasB cells, the combination did not enhance growth inhibition compared to CpdA alone (Figure 3D).

**Figure 4:**
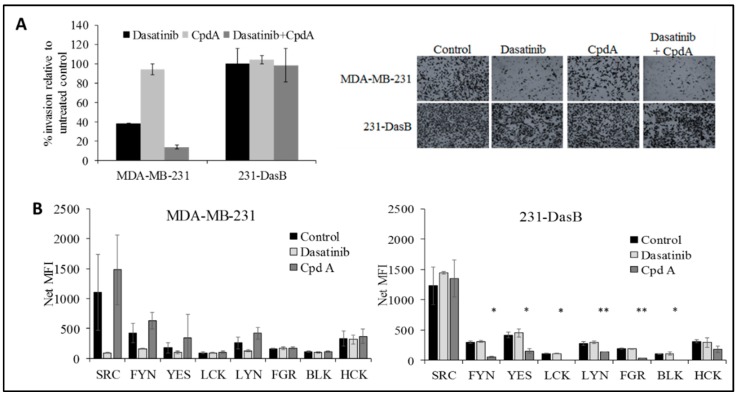
(**A**) Invasion assays in MDA-MB-231 and 231-DasB cells with/without dasatinib (100 nM) and/or CpdA (5 μM); (**B**) phosphorylation of SFKs in MDA-MB-231 and 231-DasB in response to dasatinib 100 nM and CpdA (5 µM) where NET MFI is net median fluorescence intensity. * *p* < 0.05, ** *p* < 0.01. Error bars represent the standard deviations of triplicate independent experiments. *p* values were calculated using the Student’s *t*-test.

The combined treatment also significantly reduced invasion in the MDA-MB-231 cells (*p* = 0.002), but not in the 231-DasB cells (Figure 4A). We examined the effects of CpdA on the phosphorylation of the SFKs. No significant change was observed in the parental MDA-MB-231 cells following treatment with CpdA; however, in 231-DasB cells six of the eight p-SFKs show a significant reduction in phosphorylation. FYN, YES, LCK, LYN, FGR and BLK all show significant reductions in phosphorylation in response to CpdA treatment (5 µM) (Figure 4B).

### 3.4. cMET Inhibition Blocks Dasatinib Resistance

**Figure 5:**
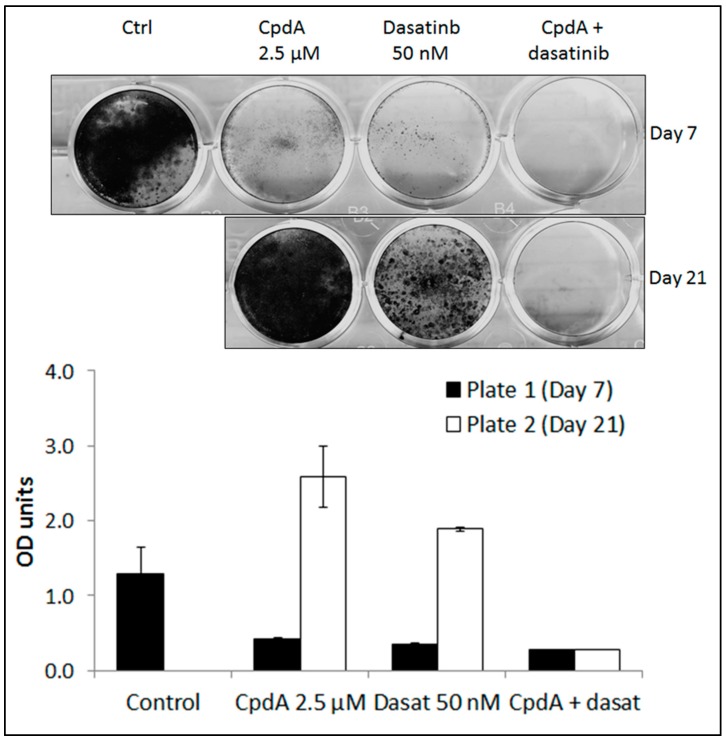
Short-term resistance assay in MDA-MB-231 cells treated with 2.5 µM CpdA and/or 50 nM dasatinib for seven days and 21 days. Optical density (OD) was determined by measuring the absorbance of the crystal violet eluted from stained cells, at 590 nM. Error bars represent the standard deviation of triplicate experiments.

To determine if cMET inhibition may prevent the emergence of resistance to dasatinib in TNBC cells, the MDA-MB-231 cells were treated with CpdA alone (2.5 µM), dasatinib alone (50 nM) or dasatinib plus CpdA. After seven days of treatment, both CpdA and dasatinib as single agents showed significant inhibition of growth (Figure 5). However, by day 21 cells treated with either CpdA or dasatinib started to grow again, suggesting the emergence of resistance, whereas the cells treated with CpdA combined with dasatinib showed no evidence of significant regrowth (Figure 5).

## 4. Discussion

Pre-clinical models of TNBC demonstrated significant sensitivity to the multi-targeted Src kinase inhibitor dasatinib (with specificity for BCR/Abl and Src family kinases); however, clinical trials with single-agent dasatinib showed limited efficacy in unselected populations [3,8,12]. In this study we sought to study the effects of long-term exposure to dasatinib on MDA-MB-231 cells, which are highly sensitive to dasatinib in three- or five-day proliferation assays [3,8]. Clinical studies indicated that significant resistance emerged within three months of continuous exposure to physiologically relevant concentrations of dasatinib [21,22]. Interestingly, we also demonstrated that resistance to dasatinib developed very quickly in the MDA-MB-231 cells. In HER2 positive breast cancer cell lines, we have found that resistance to HER2 targeted therapies generally emerges after approximately six months of continuous exposure [23,24]. This rapid development of dasatinib resistance may contribute to the lack of clinical activity observed in the single-agent phase II clinical trials.

As dasatinib is a substrate for the drug efflux pumps BCRP and MDR-1 [15], we performed accumulation assays to determine if the concentration of dasatinib achieved in the resistant cells was lower than in the parental cells. No difference in dasatinib accumulation was observed in the resistant cells and inhibition of MDR-1 and BCRP by elacridar did not enhance response to dasatinib in the resistant cells. Taken together, these results suggest that neither altered drug uptake nor efflux plays a role in the acquired dasatinib-resistant phenotype in these cells.

In the 231-DasB cells phosphorylation of Src at Y419 is not inhibited by dasatinib. This site is highly conserved across the SFKs. Therefore, to examine if specific SFKs are altered in the resistant cells we performed a multiplex assay for seven SFK members. Src was the only member of the SFK family that showed increased phosphorylation in the resistant cells. While dasatinib inhibited phosphorylation of several of the SFKs in MDA-MB-231 cells, it did not inhibit phosphorylation of any of the SFKs in the resistant cells.

Activation of Src kinase has been documented in a number of cancer types; however, activating mutations are rare. A truncating mutation in Src at Y530 has been identified in small subsets of colon and endometrial cancers [20,25]. To determine if a mutation in Src could cause constitutive Src phosphorylation in 231-DasB cells, we sequenced exons 9–12 of the *Src* gene, which encompass the kinase domain and the regulatory site at tyrosine 530 [19]. No mutations were detected in the *Src* gene the 231-DasB cell line.

Altered receptor tyrosine kinase signalling, in particular EGFR and c-Met signalling have been implicated in increased pSrc signalling in cancer, particularly in lung cancer but also in breast cancer [26,27]. Therefore, we examined whether altered RTK signalling might play a role in the dasatinib-resistant phenotype. Of the five RTKs examined, only c-MET showed an increase in phosphorylation in the resistant cells. Although EGFR has been implicated in cross-talk with both Src and MET, pEGFR levels were unaltered in the 231-DasB cells suggesting that EGFR is not a mediator of the dasatinib-resistant phenotype.

Recently another TNBC model of dasatinib resistance has been described, using the BT20 cell line. Pinedo-Carpio et al. found that chronic exposure to dasatinib resulted in increased expression of TGFβ2 and increased resistance to a TGFβ inhibitor, with a shift towards non-canonical TGFβ signalling [28]. Zhang et al. have previously shown that non-canonical TGFβ signalling can stimulate phosphorylation of Src at Tyr419 in a lung cancer cell line [29]. Thus, it is possible that non-canonical TGFβ signalling may also play a role in the dasatinib resistance in our 231-DasB model. The MDA-MB-231 cell line used in our analysis represents a post-EMT cell line [1], whilst the BT20 dasatinib-resistant model [2] is not an epithelial-mesenchymal transition (EMT) cell line. Finn et al. reported that the majority of post-EMT TNBC cell lines are highly sensitive to dasatinib, similar to MDA-MB-231. Pinedo-Carpio et al. [2] did not report any changes in cMET expression as a result of acquired dasatinib resistance, indicating that the changes we observed in cMet in the 231 DasB cells may be unique to post-EMT cells in response to dasatinib treatment. Consistent, however, with the increased p-Met levels, the 231-DasB cells also showed increased sensitivity to the Met inhibitor CpdA. Interestingly, the combination of CpdA and dasatinib resulted in significantly enhanced growth inhibition in the parental cell line but not in the 231-DasB cells. Furthermore, short-term resistance assays in the MDA-MB-231 cells showed that, in the presence of compound A, dasatinib resistance did not emerge, suggesting that c-Met inhibition may sensitise the cells to dasatinib and prevent the delay or block the development of dasatinib resistance. CpdA also enhanced dasatinib-mediated inhibition of invasion in the parental cells but not in the resistant cells. In 231-DasB cells, CpdA inhibits phosphorylation of several members of the SFK family—FYN, YES, LCK, LYN, FGR and BLK—but did not inhibit phosphorylation of Src. Our results suggest that pSrc signalling may be critical in the development of the aggressive phenotype of TNBC, and that the inhibition of p-Src may be required to overcome established resistance to dasatinib and possibly the aggressive, invasive behaviour of TNBC.

c-Met has previously been implicated in other models of acquired resistance to other small molecule inhibitors, chemotherapy and radiotherapies. Cabozantinib, a small-molecule c-Met inhibitor, has been shown to overcome gemcitabine resistance in pancreatic cancer and, quite encouragingly, displayed only a very low level of acquired resistance despite long-term treatment [30]. A similar situation was observed in two primary multiple myeloma cell lines that show increased p-Met at the development of resistance compared to the sensitive cell lines [31]. In the MDA-MB-231 cell line, ionising radiation increases the activation of c-Met and targeting the cells with a c-Met inhibitor sensitises the cells to radiation therapy [32]. Met has also been implicated in the resistance to the antiangiogenic therapy bevacizumab in glioblastomas [33]. Upregulation of c-Met was noted after exposure to bevacizumab. Our results suggest that, while c-Met may be a potential target in the acquired resistance setting, it may also be appropriate to target c-Met in combination with other therapies to prevent the development of resistance.

Dasatinib has been previously tested and has failed clinical trials in breast cancer; however, combining dasatinib with a c-Met inhibitor may be a rational therapeutic strategy to test in TNBC. Further evaluation in preclinical models of TNBC, including in vivo, would be required to support the progression of this combination into clinical trials. Furthermore, the evaluation of changes in the phosphorylation of c-Met following dasatinib treatment in tumours from patients enrolled on previous dasatinib clinical trials would be important. These trials, which included sample collection for biomarker studies ([11], NCT00817531), would elucidate whether the increase in p-Met levels that we observed in vitro following dasatinib treatment also occurs in TNBC cells in patients following dasatinib exposure. If increases in p-Met were detected in dasatinib-treated tumours, it would support clinical testing of dasatinib plus a c-Met inhibitor to block the development of dasatinib resistance.

## 5. Conclusions

In conclusion, our results suggest that cMet may be a rational target in triple-negative tumours that have developed acquired resistance to dasatinib; or, perhaps a better approach may be to combine dasatinib and cMet inhibition before metastasis occurs to improve the response to dasatinib and potentially block the emergence of resistant cells.

## Figures and Tables

**Table 1:** Doubling time in hours (± standard deviation) of MDA-MB-231 and MDA-MB-231-Das cells with and without dasatinib treatment. * indicates *p* < 0.05 calculated using the Student’s *t*-test.

Cell Line	Control	D 50 nM	D 100 nM
MDA-MB-231	17.6 ± 1.2	32.2 ± 3.3*	46.8 ± 5.1*
231 DasB	19.1 ± 2.4	21.0 ± 0.2	21.2 ± 3.1

## Supplementary Materials

The following are available online at https://www.mdpi.com/2072-6694/11/4/548/s1, Figure S1: Dasatinib accumulation assays in MDA-MB-231 parental and resistant variant cells. Cells seeded at different densities were treated with 2 µM dasatinib for two hours. Cells were then counted using the Guava Easycyte flow cytometry. The mass of dasatinib per sample was measured by mass spectrometry and expressed as ng per million cells. Error bars represent standard deviations of three replicate flasks for each seeding density, Figure S2: Proliferation assays in MDA-MB-231 parental (**A**) and 231-DasB variant (**B**) cells treated with dasatinib (Das) alone and combined with a fixed concentration of elacridar (Elac). Error bars represent the standard deviations of triplicate experiments, Figure S3: *Src* exon 9 sequence trace, Table S1: Primer Sequences for Nested PCRs.
