# Supplementary material for: Dasatinib Treatment Increases Sensitivity to c-Met Inhibition in Triple-Negative Breast Cancer Cells
Source: Cancers (Basel). 2019 Apr 17;11(4):548. doi: 10.3390/cancers11040548 (PMC6520724; doi:10.3390/cancers11040548)
Supplement: Supplementary file 1 [file cancers-11-00548-s001.pdf]

Supplementary Materials: Dasatinib Treatment Increases Sensitivity to c-Met Inhibition in Triple Negative Breast Cancer Cells

Patricia Gaule, Nupur Mukherjee, Brendan Corkery, Alex J. Eustace, Kathy Gately, Sandra Roche, Robert O'Connor, Kenneth J O'Byrne, Naomi Walsh, Michael J. Duffy, John Crown and Norma O' Donovan

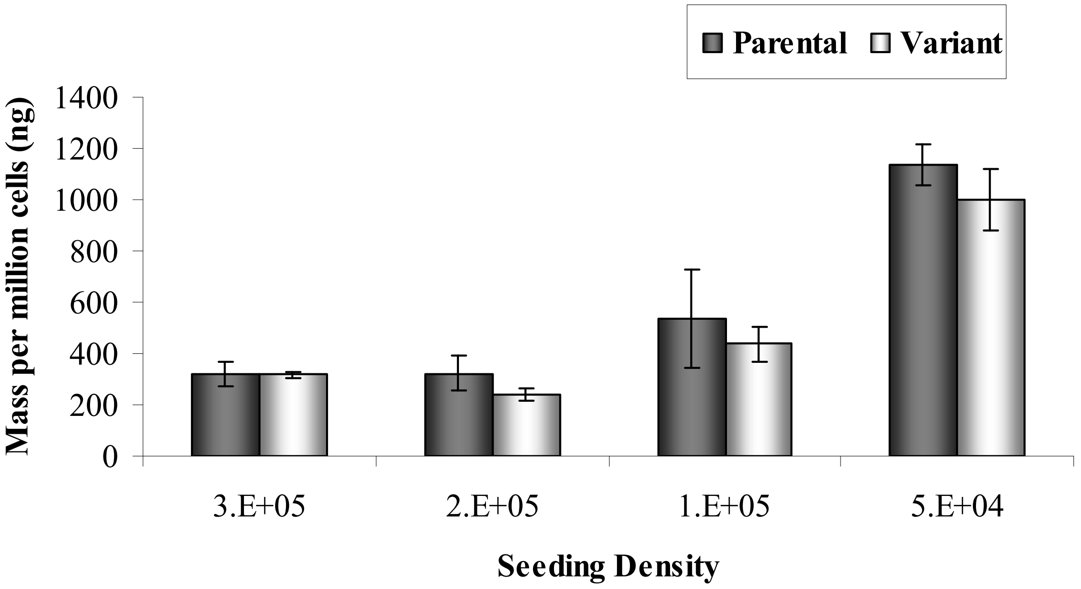

Figure S1. Dasatinib accumulation assays in MDA-MB-231 parental and resistant variant cells. Cells seeded at different densities were treated with 2 μM dasatinib for two hours. Cells were then counted using the Guava Easycyte flow cytometry. The mass of dasatinib per sample was measured by mass spectrometry and expressed as ng per million cells. Error bars represent standard deviations of three replicate flasks for each seeding density.

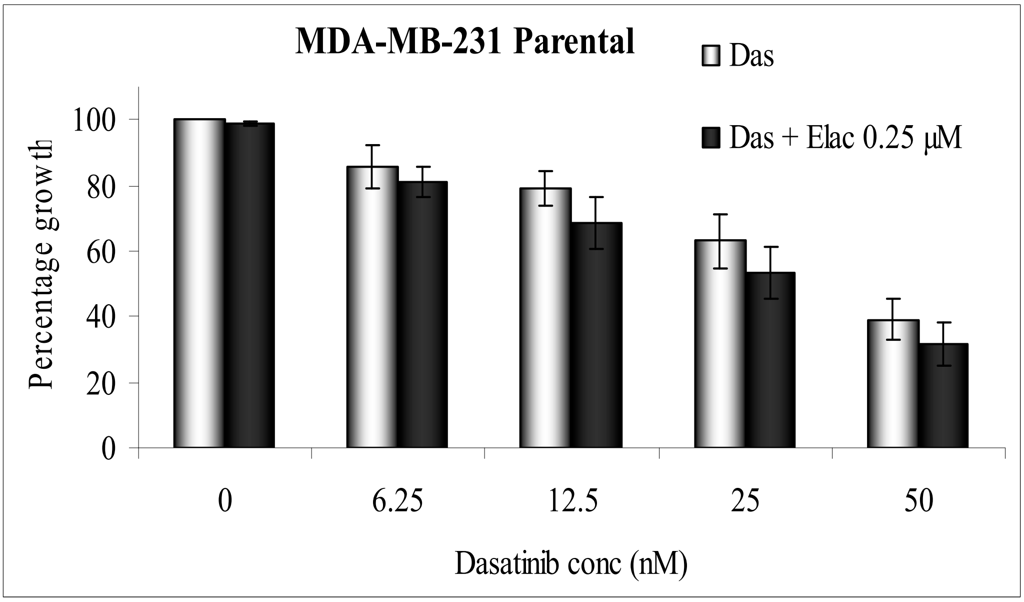

(**A**)

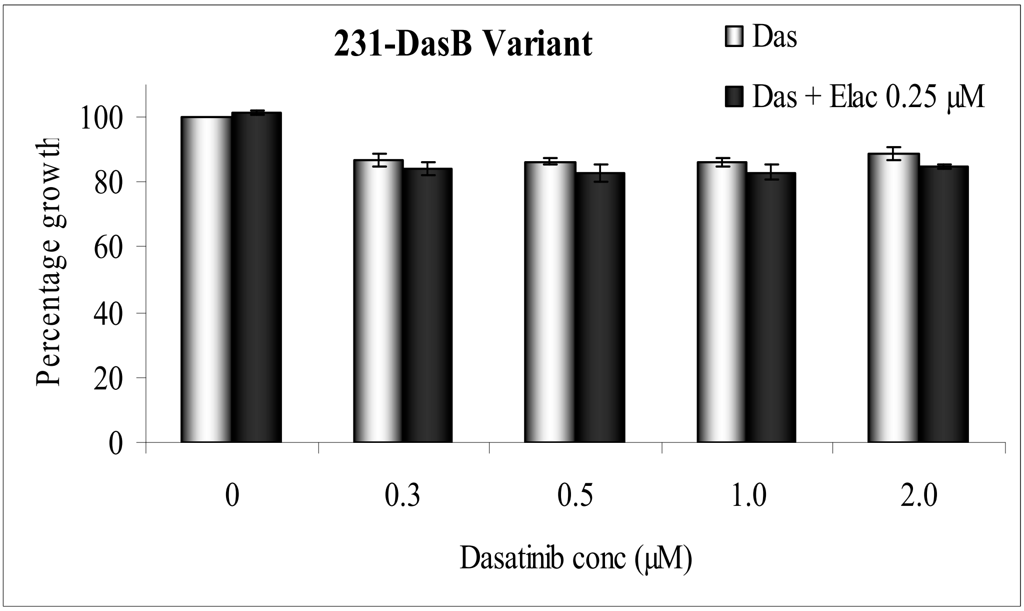

(**B**)

Figure S2. Proliferation assays in MDA-MB-231 parental (**A**) and 231-DasB variant (**B**) cells treated with dasatinib (Das) alone and combined with a fixed concentration of elacridar (Elac). Error bars represent the standard deviations of triplicate experiments.

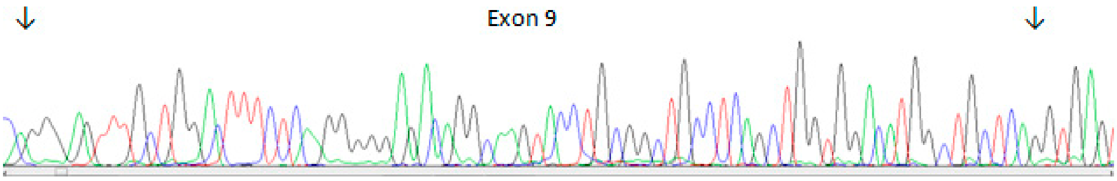

Figure S3. *Src* exon 9 sequence trace.

Table S1. Primer Sequences for Nested PCRs.

Primer Name	Primer Sequence
**SrcEXON9FOut**	5′ TGACAAGCACTGAGTGAGAC 3′
**SrcEXON9FIN**	5′ ATCACTGCATCCTGGCAGAG 3′
**SrcEXON9R**	5′ AGAACTGGCTGCACCAGATG 3′
**SrcEXON10.11FOUT**	5′ TTACTGTGAACTGACCTCAC 3′
**SrcEXON10.11FIN**	5′ TTGCCTGAAGAAGTGTGGG 3′
**SrcEXON10.11R**	5′ AGGCCATGAGGACCTTCTTA 3′
**SrcEXON12FOUT**	5′ TTGAGCGTCTGATGTTAGGC 3′
**SrcEXON12FIN**	5′ CAGGACTTATCTATGGTCACTC 3′
**SrcEXON12R**	5′ CCTGTGCCTAGAGGTTCTC 3′
